# Meaning-Centred Coping Predicted Lower Depressive Symptoms among Caregivers with Complicated Grief

**DOI:** 10.21315/mjms2024.31.5.19

**Published:** 2024-10-08

**Authors:** Chen Sung Wong, Mohamed Faiz Mohamed Mustafar, Mohd Faizal Mohd Zulkifly

**Affiliations:** 1 Department of Neurosciences, School of Medical Sciences, Universiti Sains Malaysia, Kelantan, Malaysia; 2 Hospital Universiti Sains Malaysia, Universiti Sains Malaysia, Kelantan, Malaysia; 3 Brain and Behaviour Cluster, School of Medical Sciences, Universiti Sains Malaysia, Kelantan, Malaysia; 4 Department of Psychology, Faculty of Human Development, Universiti Pendidikan Sultan Idris, Perak, Malaysia

**Keywords:** complicated grief, coping strategies, meaning-centered coping, depressive symptoms, working memory

## Abstract

**Background:**

Complicated grief is characterised by persistent low mood, intense distress and cognitive impairment. This study aimed to explore coping strategies (i.e. emotion-, problem- and meaning-centred) used by bereaved individuals facing complicated grief and how these strategies may predict psychological and cognitive outcomes.

**Methods:**

In a cross-sectional study, 20 bereaved individuals (5 males, 15 females) that aged 27 years old–65 years old (mean = 42.25, standard deviation [SD] = 9.30) were recruited following the loss of a loved one due to physical illness. Participants were screened for complicated grief and subsequently completed self-report assessments of coping strategies and depressive symptoms using Brief Grief Questionnaire (BGQ), Brief Coping Orientation to Problems Experienced (COPE) Questionnaire, Meaning-Centered Coping Scale (MCCS) and Patient Health Questionnaire-9 Items (PHQ-9). Following that, participants underwent a neurocognitive assessment of working memory using the 2-Back task.

**Results:**

Caregivers with complicated grief suffered from moderate severity of depressive symptoms (mean = 17.45, SD = 4.43) as they were coping with the losses. Furthermore, the findings showed that MCC significantly predicted lower levels of depressive symptoms (*b* = −0.50, *t* (16) = −2.25, *P* = 0.04). However, coping strategies did not significantly predict working memory performance.

**Conclusion:**

These findings highlight the potential benefits of MCC in alleviating depressive symptoms in bereaved individuals and underscore its contribution to the development of grief interventions. Grief therapists can emphasise this coping strategy to promote healing and resilience in patients in the grief work.

## Introduction

The grieving process is a natural and adaptive response to significant loss. The experience varies from person to person in terms of emotional, behavioural and social presentations. Sadness, longing, guilt and resentment are the most commonly expressed emotions. Insomnia, poor concentration and fatigue are behavioural reactions, and isolation from the social environment is the most common experience that the bereaved experience (1, 2). Although the losses associated with grief can be traumatising and disruptive, most people are able to adapt to the losses and restore functioning after a period of grief (3–5).

Nevertheless, there is a notable group of people who experience persistent feelings of grief that affect their ability to function. This type of grief response is referred to as pathological, persistent, maladaptive or complicated grief (5), which is characterised by increased and persistent psychological distress following loss. Individuals suffering from complicated grief may experience a persistent sense of disbelief and maladjustment regarding the death of a loved one. The depressed feelings, anger and resistance lead to an inability to accept the reality of the loss (6). As a result, this can prolong and intensify suffering from intense, painful emotions (7).

### Characteristics of Complicated Grief

The common symptoms suffered by caregivers with complicated grief are social withdrawal, hopelessness and self-blaming that may lead to depression (8, 9). There are several factors that affect the manifestation of depressive symptoms during grief. These include lack of social support and feelings of guilt in the face of the inevitable loss of a loved one (10).

Complicated grief is associated with lower levels of cognitive functioning, such as attention and visuospatial abilities (11). It is often accompanied by symptoms such as intrusive thoughts, rumination and avoidance behaviours, which can exacerbate cognitive distortions and processing (12). In addition, complicated grief can lead to social isolation and lower engagement in activities, limiting opportunities for cognitive stimulation and social interaction, which serve as protective factors for cognitive decline (13, 14). Neuroimaging studies have shown that total brain volumes and gray and white matter volumes are lower in bereaved individuals than in non-bereaved individuals. This may affect their functioning, as it is more difficult to maintain attention and focus on cognitive tasks when they have repeated intrusive memories and thoughts of loss (15, 16).

### Depressive Symptoms and Working Memory

Depressive symptoms and working memory impairment are two of the most important issues of complicated grief. Cognitive impairment is well documented in individuals with depressive symptoms than in healthy controls (17, 18). Meta-analyses showed that major depressive episodes have a moderate impact on neuropsychological functioning, such as deficits in attention, processing speed, memory, learning, problem-solving ability and executive functioning (19, 20). In particular, working memory is of critical importance as it is primarily involved in planning, comprehension, reasoning and problem-solving (21, 22). Moreover, it is an important mediator of functional impairment in people with major depressive disorder, as depressed individuals with poorer working memory are more likely to experience difficulties in daily living and self-care activities (23).

Individuals with higher levels of depression severity consistently performed worse on cognitive tasks related to working memory, processing speed and executive function (24, 25). In addition, other evidence suggested that anhedonia or the absence of positive affect, is associated with deterioration in perceptual speed, working memory and semantic memory in older adults with severe depressive symptoms (19, 26).

### Complicated Grief and Coping Mechanisms

According to stress coping theory (27), problem-centred coping is associated with better psychological and physiological health (28) because it helps individuals make a conscious effort to improve the situation or solve the problem. Thus, the problem-solving approach or attempts to solve the problem increases the individual’s self-efficacy and reduces vulnerability to developing depressive symptoms (29). However, it has shortcomings when it comes to dealing with adjustment stress or recent losses, which usually cannot be overcome in a short period of time (30).

Following this, emotion-centred coping is used to reduce emotional intensity or modify the emotional response to a particular event (31). Rumination and distraction are two types of emotion-centred coping (32). Rumination is more likely to maintain depressive symptoms and persistent grief symptoms (33, 34), whereas distraction may not be effective for major losses such as the death of a loved one, as it provides temporary relief and misses the opportunity to process grief (35).

### Meaning-Centred Coping in Grieving Process

Individuals may use a third type of coping, meaning-centered coping (MCC), which aims to redefine values or goals from the stressful experience to facilitate coping despite adversity (36, 37). This coping can help individuals and families reframe perceived difficulties and evaluate them as challenges or opportunities for growth rather than threats that increase psychological distress (38, 39). Specifically, MCC can improve personal coping resources by breaking the cycle of rumination and promoting better self-reflection guided by values and the meaning of life (4, 35). In addition, this type of coping can be helpful for grieving individuals as it generates positive emotions through positive reframing, finding hope, existential courage, appreciating life, and engaging in meaningful activities (40). It can maintain the upward spiral of positive emotions by valuing the negative experiences in meaningful ways, generating positive meaning, and being grateful for parts of the experiences (36). This can potentially be helpful in the case of the death of a loved one, as MCC helps maintain a positive domain of meaning, even in the presence of an intensely negative stressor.

Despite this, there has been a lack of evidence to examine the coping strategies used by people in Malaysia during complicated grief, while most studies examining the coping of grief have focused on the use of religion as a coping factor. Ahmadi and colleagues (41) examined religious coping among bereaved parents after the death of their children using a thematic analysis, while Atalay and Staneva (42) examined the internalisation of religious beliefs as a main strategy for alleviating distress and coping with loss during the COVID-19 pandemic. A more thorough examination of the various coping strategies, such as the meaning making process that underlies religious coping, could help the bereaved understand and eventually accept the reality of loss.

Thus, this study aimed to explore: i) the predictive value of coping strategies (i.e. emotion-, problem- and meaning-centred) on depressive symptoms; ii) the predictive value of coping strategies on working memory and iii) the moderating effect of MCC on the relationship between depressive symptoms and working memory.

## Method

### Design

This was a cross-sectional, correlational research.

### Participants and Sampling Method

The study recruited the participants from Hospital Universiti Sains Malaysia (HUSM), Kubang Kerian, Kelantan, through purposive sampling. Participants are selected based on the following criteria: i) Malaysians aged 18 years old–60 years old; ii) they had lost a loved one more than 6 months ago due to a physical illness such as pneumonia, cerebrovascular disease or lung disease; iii) they had been the primary caregiver for the loved one at least 3 months previously and iv) they suffered from complicated grief (Brief Grief Questionnaire [BGQ] score > 2). The exclusion criteria for the study were as follows: i) a history of psychiatric illness and ii) a history of neurological illness that impaired physical independence and/or cognitive abilities in daily living. This study also used snowball sampling, which is contacting the existing subjects to provide referrals to reach more people that fulfill the criteria of the study. The sample size estimation was calculated using G*Power 3.1.9.4. With the effect size of 0.50 and a power of 0.80, at least 27 participants were required in this study.

### Procedure

The researchers first retrieved the records of patients who had passed away due to physical illness more than 6 months ago from the Unit Record HUSM. The record was used to identify the primary caregiver. After initial contact, caregivers were contacted by phone or email. Those who expressed interest were sent a link to enroll in the study. On the first page of the enrollment form, participants received research information and a consent form. Participants then used Google Forms to fill out their demographic information and the BGQ. Eligible caregivers were invited to participate in the HUSM physically in Brain and Behavior Cluster room, School of Medical Sciences, Universiti Sains Malaysia, Kubang Kerian, by sending an invitation link to their personal contact.

On the day of the study, participants accessed the questionnaires via their personal electronic devices. They confirmed their voluntary participation and completed the items of the Brief COPE, the MCC Scale and the Patient Health Questionnaire-9 Items (PHQ-9). Participants then performed the 2-Back task in the laboratory using laptops equipped with ePRIME 2.0. During the task, participants received general instructions and a trial task before the main task. Following the trial, they performed the 2-Back task for 7 min. After completing the 2-Back task, participants were thanked for their participation and given information on how to redeem their honorarium.

The data collection process was conducted from 5 February 2023 to 30 April 2023. This study was approved by the Ethical Committee of the Universiti Sains Malaysia and participants’ informed consent was conducted in accordance with the Declaration of Helsinki.

### Materials

#### Brief Grief Questionnaire

The BGQ is a 5-item self-report or interview instrument for screening complicated grief (43). It consists of five questions about difficulty accepting death, how grief affects daily life, grief-related thoughts, avoiding loss and feeling distant from others (44). Each item was rated on a 3-point Likert scale that included the options 0 (not at all), 1 (somewhat) and 2 (a lot). Complicated grief was calculated by summing the total scores on the BGQ, with higher scores indicating increased complicated grief. Cronbach’s alpha for the BGQ was satisfactory (α = 0.75) for internal consistency, while the average variance extracted (0.39) was higher than the joint covariance (0.14) between the BGQ and the Kessler Psychological Distress Scale (K6), indicating discriminant validity between the two instruments (45). The Malaysian version of the BGQ has Cronbach’s alpha of 0.81 and a single factor loading that yielded an overall variance of 57.35%, suggesting that the scale has good internal consistency and factor loading.

#### Brief Coping Orientation to Problems Experienced

The Brief Coping Orientation to Problems Experienced (Brief COPE) is a 28-item self-report questionnaire that measures individuals’ coping processes in response to stressors. The Brief COPE has two interesting subscales that measure problem-centred coping (PCC) and emotion-centred coping (ECC) (46). Participants indicated their use of coping strategies on a 4-point Likert scale ranging from 1 (I did not do this at all) to 4 (I did this a lot). Items 2, 7, 10, 12, 14, 17, 23 and 25 were summed to score PCC, while items 5, 9, 13, 15, 18, 20, 21, 22, 24, 26, 27 and 28 were categorised under ECC. The sum of the item scores was divided by the number of items to determine the mean score of each coping form, with a higher mean score indicating greater use of each coping. The Malaysian version of the scale was validated with secondary school adolescents, with an overall Cronbach’s alpha of 0.83 (47).

#### Meaning-Centered Coping Scale

The Meaning-Centered Coping Scale (MCCS) consisted of nine items measuring individuals’ MCC. MCC is defined as the set of coping strategies used to create and maintain meaning in life in adverse situations (48). Participants indicated their agreement with nine statements about their coping mechanisms when faced with a situation, crisis or traumatic event on a 7-point Likert scale ranging from 1 (I strongly disagree) to 7 (I strongly agree). The total score was calculated by summing all nine items, with higher scores indicating better use of MCC. The MCCS has high internal consistency, with Cronbach’s alpha of 0.81–0.94 and an ICC of 0.60–0.98 across 30 countries (48). In addition, the Malaysian MCCS has an acceptable Cronbach’s alpha of 0.80 and a content validity index of 1.00 for all items rated by 10 experts in the field, indicating that the Malaysian MCCS has high relevance, clarity, simplicity and unambiguity.

#### Patient Health Questionnaire-9 Items

The PHQ-9 is a self-report instrument that assesses the severity of depression based on the nine diagnostic criteria for major depressive disorder in DSM-IV (49). Participants were instructed to indicate the frequency of specific depressive symptoms in the previous 2 weeks on a 4-point scale ranging from 0 (not at all) to 2 (most days). The higher the total score on the nine items, the more severe the individuals’ depressive symptoms. The PHQ-9 had an excellent Cronbach’s alpha coefficient of 0.92 in primary care settings and has a significant correlation with other scales measuring depression such as the Beck Depression Inventory (BAI) and the Hospital Anxiety and Depression Scale, suggesting good convergent validity (50). In Malaysia, the Malaysian version of the PHQ-9 has been validated in primary care clinics. When scored 10 and above, it has a sensitivity of 87% (95% CI: 71%, 95%) and a specificity of 82% (95% CI: 74%, 88%) (51).

#### 2-Back Task

**Figure 1:**
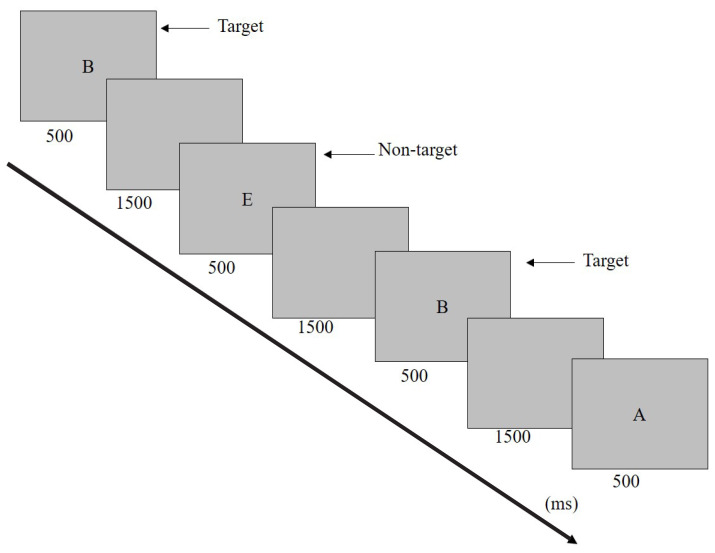
Paradigm of 2-Back task

The 2-Back task was a neuropsychological measurement of working memory via the interface of ePRIME 2.0, a software programme for designing and conducting psychological experiments. The 2-Back task involved the serial presentation of a stimulus (a series of letters from A to J) presented in a pseudorandom order. Each stimulus is presented for 500 ms and the interstimulus interval is 1,500 ms (Figure 1). The participant decides whether the current stimulus matches the one presented two trials ago and presses ‘1’ if it matches. There are 25 trials in each of the three blocks. The higher accuracy or percentage of correct matching responses (ACC) and shorter reaction time (RT) in the 2-Back task represent better working memory (52). Bootstrap correlation analyses revealed moderate to high correlations between measures (e.g. digit span sequencing and spatial span forward) expected to converge with the N-Back task (|ρ| ≥ 0.37) and weak correlations between measures (e.g. trail making test, Boston naming test) that were expected to discriminate (|ρ| ≤ 0.29), suggesting that the N-Back task has good convergent and discriminant validity (53). Figure 1 shows the paradigm of the 2-Back task.

### Statistical Analyses

Statistical analyses were performed using IBM SPSS Statistics version 26.0. The sociodemographic characteristics of the subjects were summarised using descriptive statistics, with numerical data presented as mean and standard deviation (SD) based on their normal distribution. To achieve objectives 1 and 2, i.e. to examine the predictive value of coping strategies on depressive symptoms and working memory, multiple regression analysis was performed using the forced entry method. Each predictor was entered simultaneously and a model with a *P*-value of < 0.05 was considered a significant predictor of outcome. Beta weights were used to assess the relative strength of the predictors in predicting the outcome and Cohen’s *f*2 was used to determine the effect size of the multiple regressions. For Objective 3, examining potential moderating effects, Model 1 of PROCESS Macro v4.3 in SPSS was used (54), with a *P*-value of < 0.05 indicating a significant interaction between predictor and moderator, suggesting the presence of moderation.

## Results

### Demographic Details

The respondents were males (*n* = 5) and females (*n* = 15). There were Malays (*n* = 19) and Chinese (*n* = 1). Most of the participants were caregivers of deceased patients with pneumonia (*n* = 6), followed by pulmonary disease (*n* = 5), cerebrovascular disease (*n* = 4), cardiac disease (*n* = 4) and heart disease (*n* = 1). Participants’ ages ranged from 27 years old to 65 years old (mean = 42.25, SD = 9.30 years old). Table 1 shows the demographic distributions.

### Descriptive Statistics

Participants scored a mean PHQ-9 of 17.45 (mean = 17.45, SD = 4.43), indicating that participants had moderately severe depressive symptoms. In working memory performance, the 2-Back task showed that participants had a mean ACC of 14.07% (SD = 12.61) and RT of 411.17 ms (SD = 38.42).

### Correlation Analysis

Correlation analysis using Pearson’s correlation coefficient (*R*) showed that there was only one significant relationship, negative correlation between MCC and depressive symptoms, while the other relationships were not significant, *P* > 0.05. Table 2 shows the correlations between the variables.

### Regression Analysis

This study aimed (1) to determine how coping strategies can predict the severity of depressive symptoms. The overall model of coping strategies explained 34.2% of the variance in depressive symptoms, *R^2^* = 0.34, *F* (3, 16) = 2.78. Specifically, MCC significantly predicted depressive symptoms when controlling for PCC and ECC (*b* = −0.50, *t* (16) = −2.25, *P* = 0.04). Conversely, PCC did not significantly predict depressive symptoms when controlling for ECC and MCC (*b* = −0.06, *t* (16) = −0.22, *p* = 0.83). Finally, ECC did not significantly predict depressive symptoms when controlling for PCC and MCC (*b* = 0.41, *t* (16) = 1.48, *P* = 0.16). Table 3 shows the regression analysis for the model of coping strategies and depressive symptoms.

This study aimed (2) to assess the extent to which coping strategies can predict working memory, as indicated by ACC and RT. For ACC, the overall model that included coping strategies predicted 19.8% of the variance in ACC (*R^2^* = 0.20, *F* (3, 16) = 1.32) but MCC did not significantly predict accuracy in ACC when controlling for PCC and ECC (*b* = −0.32, *t*(16) = −1.32, *p* = 0.21). Similarly, PCC did not significantly predict ACC when controlling for ECC and MCC (*b* = −0.03, *t(*16) = −0.50, *P* = 0.63). Subsequently, ECC did not significantly predict ACC when controlling for PCC and MCC (*b* = 0.40, *t*(16) = 1.29, *P* = 0.22). Table 4 shows the regression analysis for the model of coping strategies and ACC.

For RT, the overall model, which included the predictors PCC, ECC and MCC, predicted 20.3% of the variance in RT (*R^2^* = 0.20, *F* (3, 16) = 1.36). MCC did not significantly predict accuracy in RT when controlling for PCC and ECC (*b* = 0.26, *t*(16) = 1.06, *P* = 0.30). Similarly, PCC did not significantly predict accuracy in RT when controlling for ECC and MCC (*b* = 0.03, *t*(16) = 0.11, *P* = 0.92). Finally, ECC did not significantly predict RT when controlling for PCC and MCC (*b* = −0.43, t *(*16) = −1.41, *P* = 0.18). Table 5 shows the regression analysis for the model of coping strategies and RT.

### Moderation Analysis

For the third objective, the overall model consisting of depressive symptoms, MCC and the interaction term between depressive symptoms and MCC did not predict ACC and RT (*R^2^* = 0.11, *F* (3, 16) = 0.68, *P* = 0.58) and depressive symptoms (*R^2^* = 0.17, *F* (3, 16) = 3.28, *P* = 0.05). Following that, depressive symptoms and MCC did not significantly predict ACC (*b* < 0.00, *t*(16) = 0.21, *P* = 0.83) and RT (*b* = −0.18, *t*(16) = −1.17, *P* = 0.27). Thus, it can be inferred that MCC did not significantly moderate the relationship between depressive symptoms and working memory (ACC and RT). Tables 6 and 7 show the moderation analysis for ACC and RT, respectively.

## Discussion

A key finding of this study was that MCC predicted significantly lower depressive symptoms in the bereaved individual than in the non-bereaved individual. We also demonstrated that caregivers with complicated grief exhibited moderate levels of depressive symptoms such as persistent and intense grief reactions, lack of social support, and feelings of guilt, supporting the findings of previous studies (7). However, we show that all coping strategies in this study did not predict the working memory performance of caregivers with complicated grief. Moreover, this study showed that MCC did not moderate the relationship between depressive symptoms and working memory performance.

### Coping Strategies and Depressive Symptoms

The findings showed that the first hypothesis was supported as MCC significantly predicted lower depressive symptoms among bereaved individuals than PCC and ECC. This is consistent with the theoretical framework of broaden-and-build theory that proposed that MCC broaden people’s momentary thought-action repertoires, which in turn enhances the individuals’ social, emotional, and cognitive resources (55, 56). Over time, these enduring personal resources serve as building blocks for increased well-being and resilience (57). Furthermore, MCC can create an upward spiral of positive emotions that build up their coping resources for handling future adversities and break the cycle of negative downward spiral of depressive symptoms (58, 59). As one of the major challenges confronted by individuals with prolonged grieving, the upward spiral could possibly reduce emotional suffering and cope with intense emotions, which will reduce the vulnerability to depressive symptomatology in the long run (59, 60).

MCC may outperform PCC as a predictor of depressive symptoms during complicated grief, as PCC can only lead to improved adaptation to stressors that can be controlled and modified (30). Certainly, the nature of complicated grief is associated with stressors that are both uncontrollable and protracted, which reduces the effectiveness of PCC to alleviate emotional pain (37). In addition, mourners may rely less on PCC, which involves active problem-solving strategies that require higher cognitive resources. As a result, depletion of cognitive resources in coping with long-term stressors, such as coping with complicated grief, could lead to greater burnout and psychological distress (61).

As for ECC, it can lead to mixed results when it comes to coping with depressive symptoms and complicated grief (62). In particular, the use of avoidance strategies can provide temporary relief by pushing unwanted emotions and thoughts out of consciousness, but it can exacerbate depressive symptoms over time (32). This is because the temporary effects can be particularly problematic when an avoidant coping strategy manifests in substance use, risk-taking behavior, or social withdrawal (63). Alternatively, some people may use ECC as a means to openly express and process their emotions related to grief, which promotes a sense of emotional catharsis. While the expression of emotions is crucial, relying solely on ECC can intensify emotional experiences and lead to increased rumination and depressive thoughts (34).

Finally, considering that the primary sample groups were Malay Muslims, it follows that both religiosity and culture could influence the selection and implementation of coping strategies. Religious beliefs provided a framework for understanding and interpreting the meaning of life events, including the loss of loved ones. For believers, the idea that their lives have been blessed by a higher being can provide reassurance and comfort in times of distress (64). The belief that there is a purpose or greater plan behind their suffering can provide a sense of purpose and hope and make the burden of loss more bearable (41). In addition, believing that the negative events they experience are opportunities for spiritual growth and development can promote resilience and encourage people to seek positive lessons or personal strengths (65). As a result, they are more likely to use MCC in coping with grief and loss.

### Coping Strategies and Working Memory Performance

In addition, the second hypothesis was not supported, as coping strategies did not significantly predict working memory performance in grieving individuals. There are several explanations for why coping strategies did not predict working memory performance in this study. First, PCC directly addresses the practical aspects of the stressor by organising and managing the tasks associated with the loss. However, the cognitive demands associated with working memory tasks, such as retrieving and mentally processing information, may not be directly affected by these problem-solving strategies. Even if individuals can effectively manage the practical aspects of their grief, this is not necessarily predictive of significant changes in their working memory performance (31). ECC is often aimed at regulating emotions rather than directly influencing cognitive processes such as working memory. If emotional distress does not directly affect cognitive load on working memory, there may be no predictive values (66). Finally, the effects of MCC may be reflected in long-term psychological well-being rather than immediate cognitive performance (37). Working memory tasks, which are more immediate, may not capture the delayed effects of meaning making.

In addition, it is possible that coping strategies are a consequence rather than a cause of working memory performance (67). That is, individuals with better working memory performance may be better able to use coping strategies effectively. Prussien and colleagues (68) found that individuals with higher executive function use more secondary coping strategies because they are able to divert more cognitive resources into active cognitive coping than individuals with lower attentional resources and poor working memory. This implies that an intact working memory is one of the determinants of the use of coping strategies that require cognitive control and deliberative cognitive processing. Mindfulness-based coping, with its focus on attentional control in the present moment, can reduce distractions and rumination while improving cognitive flexibility (69). These results enable individuals to manage cognitive demands more effectively, leading to improved cognitive functions such as working memory and executive functions (70).

### Moderating Effect of MCC and Depressive Symptoms

Our findings showed that MCC does not moderate the relationship between depressive symptoms and working memory can be explained by the reverse directional effects of cognitive function, particularly working memory, on the use of coping strategies. A mediation analysis (68) suggested that cognitive dysfunction may partially lead to depression through its effects on coping. Specifically, the study indicated that cognitive deficits may hinder individuals’ ability to use adaptive coping mechanisms, which may increase the likelihood of developing depressive symptoms. Therefore, an individual’s cognitive performance could be a potentially more important moderator in moderating the relationship between coping strategies and depressive symptoms in survivors. For example, individuals with high cognitive performance engaged in cognitive structuring that reduced depressive symptom severity than those with low cognitive performance.

Furthermore, it is hypothesised that the effects of MCC may be more noticeable in older people, as those affected by complicated grief show greater cognitive decline than those without complicated grief, even when preexisting cognitive abilities are taken into account (71). Consequently, the study of MCC in the context of older adults may provide more valuable insights into the potential moderating effects of this coping mechanism. Furthermore, the effectiveness of MCC may be influenced by the severity of complicated grief. This suggests that study participants may be individuals who experience a milder or shorter duration of grief following a loss compared to others. Consequently, many mourners in the sample could be going through the typical grieving process without actively using specific coping strategies (72). In simpler terms, the varying degree of grief intensity within the sample might influence how well MCC moderates the relationship between depressive symptoms and working memory.

### Implications

The findings of this study are particularly useful in adding to the literature on coping strategies in grief studies (26, 73). The findings provided empirical evidence to support the inclusion of MCC with the ‘M’ in the PERMA (Positive Emotion, Engagement, Relationships, Meaning, and Achievement) framework of positive psychology that encompasses five essential elements contributing to well-being: i) positive emotions, ii) engagement, iii) relationships, iv) meaning and iv) accomplishments (74). These paradigms emphasise the importance of integrating both the positive and negative aspects of life and propose transforming suffering into personal growth through meaning. Thus, positive existential therapy offers a potential approach to interventions that use MCC as compared to traditional grief interventions that emphasise on emotional processing and reframing of negative thought patterns (75).

### Limitations and Future Recommendations

This study has some limitations. First, due to the correlational design of this study, it is only possible to establish causality between the variables to a limited extent. Therefore, the results should be interpreted with caution. Future longitudinal studies should track changes in depressive symptoms over time to establish causality and identify the course of depressive symptoms in response to different coping strategies. In addition, experimental studies may be proposed to examine the efficacy of a meaning-centred intervention such as Breitbart’s module of meaning-centred psychotherapy (76). To address the small sample size (*n* = 20) in this study, researchers may collaborate with multiple institutions (e.g. peer grief support groups and hospice centres) that help increase the sample size by pooling data from multiple sources. Finally, the fact that this study relied on self-report questionnaires (BGQ, Brief COPE, MCCS, PHQ-9) as the primary method of data collection may limit the validity of the results. Self-report questionnaires are subject to potential biases such as social desirability and recall error, which may affect the accuracy and reliability of the results (77). The use of a clinical diagnostic interview such as Structured Clinical Interview for DSM-V and behavioural observation may provide more robust results on the predictive value of coping strategies in outcome measures of depressive symptoms.

## Conclusion

Overall, the results of this study showed that caregivers with complicated grief suffered from moderate to severe depressive symptoms as they processed the loss and adjusted to life without their loved one. Therefore, this work examined the predictive value of three types of coping, namely, problem-, emotion- and meaning-centered, on depressive symptoms and working memory performance. Consistent with broaden-and-build theory, MCC predicted lower depressive symptoms in bereaved individuals. In contrast to other coping strategies, problem- and emotion-centred coping did not predict depressive symptoms during the complicated grieving process. However, the predictive effects for working memory were not significant, as none of the coping strategies significantly predicted working memory performance. Finally, there were no moderating effects of MCC on the relationship between depressive symptoms and working memory. The findings of this study can be added to the literature on coping strategies, depressive symptoms and working memory in family caregivers with complicated grief in the context of Malaysia. This could help researchers or practitioners in developing a framework or module for effective grief intervention for those with greater vulnerability to psychopathology.

## Figures and Tables

**Table 1:** Demographic distribution

Descriptors	Characteristics	*n*	%	mean	SD
Gender	Male	5	25.0	–	–
	Female	15	75.0	–	–
Ethnicity	Malay	19	95.0	–	–
	Chinese	1	5.0	–	–
Caregivers to patient with	Pneumonia	6	30.0	–	–
	Cerebrovascular diseases	4	20.0	–	–
	Lung diseases	5	25.0	–	–
	Heart diseases	4	20.0	–	–
	Cancer	1	5.0	–	–
Age		–	–	42.25	9.30

Note: SD = standard deviation

**Table 2:** Correlations between variables

	PCC	ECC	MCC	PHQ-9	ACC	RT
PCC	–					
ECC	0.67	–				
MCC	0.38	0.16	–			
PHQ-9	0.02	0.29	−0.46*	–		
ACC	−0.02	0.23	−0.32	0.17	–	
RT	−0.16	−0.36	0.20	−0.34	−0.32	–

Notes:

* *P* < 0.05 level (2-tailed);

PCC = Problem-Centred Coping; ECC = Emotion-Centred Coping; MCC = Meaning-Centred Coping; PHQ-9 = Patient Health Questionnaires-9 Items; ACC = accuracy; RT = reaction time

**Table 3:** Regression analysis for model of coping strategies and depressive symptoms

	Unstandardised coefficient		Standardised coefficient	*t*	*P*-value

	*b*	Standard error	Beta		
(constant)	20.19	6.37		3.17	0.006
PCC	−0.45	2.07	−0.06	−0.22	0.830
ECC	4.15	2.81	0.41	1.48	0.158
MCC	−0.21	0.10	−0.50	−2.25	0.039*

Notes:

* *P* < 0.05; PCC = Problem-Centred Coping;

ECC = Emotion-Centred Coping; MCC = Meaning-Centred Coping

**Table 4:** Regression analysis for model of coping strategies and accuracy (ACC)

	Unstandardised coefficients		Standardised coefficients	*t*	*P*-value

	*b*	Standard error	Beta		
(constant)	0.17	0.20		0.85	0.411
PCC	−0.03	0.07	−0.16	−0.50	0.626
ECC	0.11	0.09	0.40	1.29	0.215
MCC	−0.00	0.00	−0.32	−1.32	0.207

Notes: PCC = Problem-Centred Coping; ECC = Emotion-Centred Coping; MCC = Meaning-Centred Coping

**Table 5:** Regression analysis for model of coping strategies and reaction time (RT)

	Unstandardised coefficients		Standardised coefficients	*t*	*P*-value

	*b*	Standard error	Beta		
(constant)	444.31	60.81		7.31	0.000
PCC	2.09	19.78	0.03	0.11	0.917
ECC	−37.75	26.81	−0.43	−1.41	0.178
MCC	0.96	0.91	0.26	1.06	0.304

Notes: PCC = Problem-Centred Coping; ECC = Emotion-Centred Coping; MCC = Meaning-Centred Coping

**Table 6:** Moderation analysis (model of accuracy)

	Coefficient	Standard error	*t*	*P*-value
(constant)	0.52	0.93	0.56	0.584
PHQ-9	−0.01	0.04	−0.19	0.854
MCC	−0.01	0.02	−0.44	0.667
Int_1	0.00	0.00	0.21	0.834

Notes: PCC = Problem-Centred Coping; ECC = Emotion-Centred Coping; MCC = Meaning-Centred Coping

**Table 7:** Moderation analysis (model of reaction time)

	Coefficient	Standard error	*t*	*P*-value
(constant)	253.66	222.17	1.14	0.270
PHQ-9	6.31	9.10	0.69	0.498
MCC	4.00	3.74	1.07	0.300
Int_1	−0.18	0.15	−1.17	0.259

Notes: MCC = Meaning-Centred Coping; PHQ-9 = Patient Health Questionnaires-9 Items
